# Prohibitin-1 cysteine-69 plays a sex-dependent role in fatty acid metabolism

**DOI:** 10.1016/j.jlr.2026.101158

**Published:** 2026-09-23

**Authors:** Katherine J. Bernier, Zhanguo Gao, Yeshika Bhatia, Niloofar Beheshti Dehkordi, Fatemeh Nasiri, Alexes C. Daquinag, Renata Pasqualini, Wadih Arap, Mikhail G. Kolonin, Suresh Mishra

**Affiliations:** 1 Department of Physiology and Pathophysiology, Faculty of Health Sciences, University of Manitoba, Winnipeg, Manitoba, Canada; 2 Brown Foundation Institute of Molecular Medicine, McGovern Medical School, The University of Texas Health Science Center at Houston, Houston, TX, USA; 3 Department of Internal Medicine, Faculty of Health Sciences, University of Manitoba, Winnipeg, Manitoba, Canada; 4 Division of Cancer Biology, Rutgers Cancer Institute, Newark, NJ, USA; 5 Division of Cancer Biology, Department of Radiation Oncology, Rutgers New Jersey Medical School, Newark, NJ, USA; 6 Division of Hematology and Oncology, Department of Medicine, Rutgers New Jersey Medical School, Newark, NJ, USA

**Keywords:** brown adipose tissue, white adipose tissue, fatty acid transport, mitochondria, glucose metabolism, sex differences

## Abstract

Metabolic diseases are caused by dysregulation of glucose and lipid homeostasis, which relies on the function of adipose tissues (ATs). This regulation and the associated pathologies differ between sexes, which remain incompletely understood. Prohibitin-1 (PHB1) has multifaceted tissue-specific functions converging on metabolism. Here, we investigated the role of PHB1’s evolutionarily conserved cysteine-69 by replacing it with an alanine in a knock-in (*Phb1-*KI) mouse model. We show that *Phb1-*KI mice lack cell surface localization of PHB1 and display sex-biased AT alterations in relative sizes of visceral and subcutaneous depots and their adipocyte sizes, expression of lipid metabolism effectors, and adipokine secretion. *Phb1-*KI adipocytes are defective in long-chain fatty acid uptake and mobilization, and their mitochondrial abnormalities and reduced oxidative capacity result in defective lipid metabolism. In compensation, *Phb1-*KI have increased glycolysis and *Phb1-*KI mice display improved glucose disposal and increased insulin sensitivity. Attributes of this phenotype manifested differently in males and females of *Phb1-*KI mice. Our results demonstrate the importance of cysteine-69 in sex-related function of PHB1 in integrative physiology and systemic metabolism.

## Graphical abstract

**Figure:**
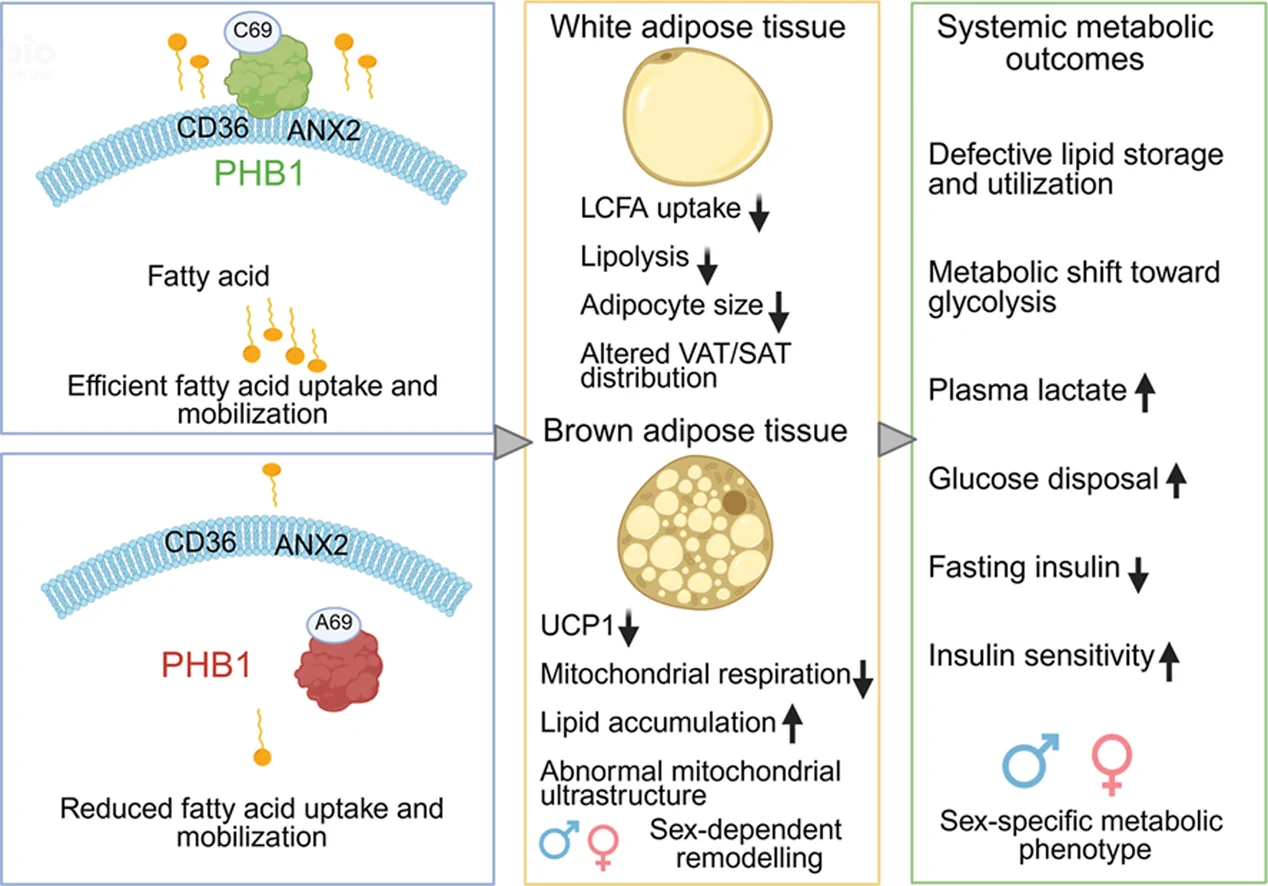

Metabolism relies on glucose and lipid homeostasis, which is disrupted in obesity-associated metabolic disease (1, 2). Adipocytes in adipose tissue (AT), integral to maintaining energy balance (3), are of two major types (4). Adipocytes in white AT (WAT), present as visceral (VAT) and subcutaneous (SAT) depots, store energy as triglycerides in unilocular lipid droplets (5). WAT distribution and function vary during growth and aging, which differ between biological sexes. Accordingly, WAT-related disorders, including obesity, type 2 diabetes, and metabolic syndrome, often exhibit sex-related differences in risk and etiology (6). Brown AT (BAT), localized in the interscapular, cervical, and supraclavicular regions, contains adipocytes with multilocular lipid droplets and numerous mitochondria (7, 8). Brown and beige adipocytes, also inducible in WAT, express uncoupling protein 1 (UCP1) enabling nonshivering thermogenesis (9, 10, 11). The amount and activity of thermogenic AT also differ between sexes: females typically display its higher activity, linked with higher resistance to type 2 diabetes development (12). Although sex steroids are known to affect adipose biology during different stages of life (e.g., puberty, pregnancy, and aging), as well as during gender-affirming hormone therapy, the cellular and molecular mediators and the underlying mechanisms that connect hormonal input to metabolic outcomes in AT remain incompletely understood.

Prohibitin-1 (PHB1) is an evolutionarily conserved protein with multiple functions spanning different cellular compartments, including the mitochondria, plasma membrane, and the nucleus (13, 14, 15, 16, 17, 18). In vitro and in vivo gain-of-function and loss-of-function models of PHB1 have established a key role in the coordinated regulation of lipid homeostasis in the body together with different functions in distinct types of adipose cells (17, 19, 20, 21, 22). Specifically, in an adipocyte-specific *Phb1* KO, white adipocytes displayed defective long-chain fatty acid (LCFA) uptake and lipolysis, whereas brown adipocytes had reduced mitochondrial function and thermogenic capacity (20), indicating cell type-specific function of PHB1 in AT. The BAT/WAT function balance driving lipid utilization is important in the reciprocal regulation of glucose metabolism, dysregulated in type 2 diabetes (23). We have reported that the inactivation of *Phb1* in adipocytes, reducing fatty acid utilization, improves glucose tolerance at the organismic level (19, 20). Active sites of PHB1 involved in its pleiotropic functions in different types of adipocytes have not been established. PHB1 undergoes S-acylation (e.g., palmitoylation) at the Cys^69^ site in adipocytes, which facilitates its protein-protein interactions and membrane anchoring (24). Several proteins have been shown to bind PHB1, including Annexin A2 (ANX2) (25), CD36 (26), and Eps 15 homology domain protein 2, all of which regulate lipid transport (24). Mouse knockouts of *Phb1*, *Cd36*, and *Anx2* have overlapping phenotypes, indicating the importance of this translocase complex in LCFA intercellular transport in WAT (27).

Cysteine has a highly ionizable and oxidation-sensitive thiol (-SH) group that can undergo a wide variety of oxidative posttranslational modifications (PTMs) (28). Such diversity can contribute to cell type-specific functions and may also contribute to sex-related differences. For example, sex differences in mitochondrial ROS production in different tissue types (28) may involve differential modification at cysteine residues in target proteins in the mitochondria. (29). In PHB1, there is a single cysteine residue at position 69 (Cys^69^) in human and mouse that is present in the PIIFDCRSRPR motif, which is conserved in vertebrates (from zebrafish to human).

Here, we hypothesized that the Cys^69^ of PHB1 is important for its role in AT biology and metabolism. To test this hypothesis, we created a strain of *Phb1*^C69A^ knock-in (*Phb1-*KI) mice in which Cys^69^ is replaced with alanine (Cys69Ala) in vivo. Here, we report that Cys^69^ is required for PHB1 localization to the cell surface, for the function of PHB1 in promoting LCFA trafficking in and out of white adipocytes, and for normal mitochondrial function in BAT and skeletal muscle. We provide evidence that Cys^69^ serves different functions in distinct cellular compartments in a sex-biased manner and regulates AT and systemic metabolism.

## Materials and methods

### Generation of *Phb1* KI mice

The *Phb1*-KI mouse model in the C57BL/6J strain was developed in-house at the Genetic Modeling Center at the University of Manitoba, Canada. The gRNA targeting the *Phb1* gene within the mouse genome, along with the donor vector carrying a knock-in model cassette and Cas9 mRNA were injected together into fertilized eggs to generate the desired genetic modification in the resultant offspring. The *Phb1* knock-in mouse model had three main changes: the cysteine residue on position 69 was replaced with an alanine residue, a Hae III restriction enzyme site was inserted for genotyping purposes, and the PAM (protospacer adjacent motif) site was disrupted upon donor sequence integration to prevent reediting (supplemental Fig. S1A). Genotypes were confirmed by isolating and amplifying DNA from ear punch samples by PCR, then subjecting the DNA to Hae III digestion followed by gel electrophoretic analysis (supplemental Fig. S1B). The resultant mice were identified by a PCR and confirmed by DNA sequencing.

### Animal experiments

All animal experiments were conducted as per the Animal Use Protocols 20-008/1/2/3 and 24-023 approved by the University of Manitoba and Protocol AWC-21-0145 approved by the UT Houston IACUC. Genotyping of the *Phb1*-KI mice was performed by using ear-punch samples. Experimental animals were housed in a controlled environment with a 12-h light, 12-h dark cycle at 22 °C with a standard chow diet (Lab Diet, St. Louise, MO) and water ad libitum. 12-week-old males and females *Phb1*-KI mice and their age and sex matched WT control mice were used for the study. High-fat diet (HFD) (60% kcal % fat; D12492) was purchased from Research Diets (New Brunswick, NJ). Male and female *Phb1*-KI and WT control mice were fed an HFD or chow diet for 16 weeks, and body weights were recorded on a weekly basis.

### Fatty acid uptake and mobilization

Lipolysis was induced by intraperitoneal injection of isoproterenol (10 mg/kg). Plasma FFA were measured with a kit from BioAssay System (EnzyChromTM Free Fatty Assay Kit EFFA-100). BODIPY-C16 (Molecular Probes, Waltham, MA) was injected iv (0.1 mg). For ex vivo uptake experiments, BODIPY-C16 (2 μM) was added to cells. Transwell assay was setup and data were analyzed as previously described (19).

### Phage peptide PHB1 homing assays

In vivo tissue distribution of PHB1-binding cyclic peptide CKGGRAKDC displayed on bacteriophage was performed as described (19, 30). Then, tissues were recovered, and transforming units per gram of tissue was quantified by K91 host *E. coli* infection and tetracycline resistance as described (31). For ex vivo uptake experiments, 10^9^ transforming unit of CKGGRAKDC-phage was added to cells and incubated for 1 h prior to washing in PBS containing 1 mM MgCl2/1 mM CaCl2 for cell lysis, and K91 host *E. coli* infection for quantification as described (32).

### Blood and tissue collection

Blood, serum and AT samples from homozygous male and female *Phb1*-KI mice and C57BL/6J WT mice of about 6 months of age were collected as described (19, 21). Fasting plasma glucose, insulin, and lactate were measured as we described previously (33, 34).

### Glucose and insulin tolerance tests (ITTs)

Glucose tolerance test (GTT) and insulin tolerance test (ITT) were performed as described previously (19, 21) Blood glucose concentrations were measured using a handheld glucometer with glucose test strips according to the manufacturer’s instructions.

### Histological analysis

WAT and BAT samples from 3-month-old mice were fixed in 10% buffered formalin. Samples were then dehydrated and embedded in paraffin blocks. A total of 5 μm sections on each slide were stained with H&E. Sections were imaged at 40X magnification by using an Evos XL Core Imaging System. Images were analyzed by using ImageJ software (NIH) with the Adipocytes Tool macro downloaded as described previously (11). All 40-fold images were analyzed with Adiposoft-ImageJ, a fully automated open-source software for the analysis of WAT cellularity in histological sections (35).

### Transmission electron microscopy (TEM)

The TEM analysis of BAT from different experimental groups was performed by using a Philips CM10 at 80 kV (19). Briefly, the BAT tissue samples were excised into approximately 1 mm^3^ and fixed with 3% glutaraldehyde in 0.1 M Sorensen’s buffer for 3 h. After fixation, the tissues were resuspended in 5% sucrose in 0.1 M Sorensen’s buffer and then embedded in EPON™ resin. Subsequently, analysis was performed on ultrathin sections (100 nm) stained with uranyl acetate and counterstained with lead citrate.

### Cell culture

For stromal cell isolation, outer ears, or SAT pads of 8-week-old mice were excised, minced, and digested in 0.5 mg/ml collagenase type I (Worthington Biochemical) and 2.5 mg/ml dispase Roche, 04942078001) solution in a shaking bath for 1 h at 37°C. The cell suspension was filtered through a 70 μm cell strainer (Thomas Scientific, cat # 1181X53) followed by centrifugation (360g, 5 min, RT). 3T3-L1 cells were from ATCC. For white adipogenesis induction, cells grown to confluence were cultured in medium containing 1.7 μM insulin/0.5 mM IBMX, 1 μM dexamethasone, and 5 μM pioglitazone for 3 days and 1.7 μM insulin afterward, as previously described (20).

### Mitochondrial respiration analysis

BAT or quadriceps muscles were minced and mixed evenly in a Petri dish on ice. 70 mg of tissues were loaded into a 1.5 ml Eppendorf tube on ice. A total of 700 μl cold Seahorse assay medium was added into the tubes and homolyzed with Kimble Pellet Pestle Cordless Motor (cat. # 749540) with Blue Pestle Attachment (1.5 ml Microtube size, cat. # 749520) on ice for several minutes until no debris was observed (0.1 mg/ul). A total of 50 μl of homolysis (5 mg tissue) were loaded into the center of XF24 Islet Capture Microplate (Agilent 101122-100). The Seahorse XF Cell Mito Stress Test Kit (Agilent Technologies, 103015-100) was used to analyze mitochondrial oxidation. Oxygen consumption rate (OCR) was measured upon successive treatment with oligomycin (2.1 μM), FCCP (Carbonyl cyanide-p-trifluoromethoxyphenylhydrazone, 8.4 μM), and rotenone/antimycin A (5.2 μM). For the palmitate oxidation stress test, 200 μM of palmitate-BSA (Agilent, cat. # 102720) was used in Seahorse medium, and Mito-stress assay was conducted.

### Immunoassays

For immunoprecipitation (tissue was homogenized in RIPA buffer supplemented with protease inhibitor cocktail (Roche, #04574834001). Protein lysates were clarified by centrifugation at 12,000g for 15 min at 4°C. Immunoprecipitation was performed as previously described (27). Briefly, cleared supernatants were precleared with Protein A/G beads for 1 h at 4°C before incubation with anti-PHB antibody (MilliporeSigma, #HPA003280) used at 5 μg IgG per mg of protein extract.

Tissue samples from mice were analyzed by Western blotting as described previously (11, 19). Tissue lysates or immunoprecipitates were separated on 4%–20% gradient SDS-PAGE gels and transferred to Immobilon-FL membranes (MilliporeSigma, #IPFL00010). Membranes were blocked with Odyssey Blocking Buffer (LI-COR, #927-70001) and incubated with anti-PHB or anti-CD36 (R&D Systems, #AF1955) antibodies. Immunoreactivity was detected using the Odyssey CLx Imaging System (LI-COR).

For immunofluorescence, mouse ear fibroblasts induced for white adipogenesis for 6 days were used. Upon treatment with 0.3 mM palmitic acid for 10 min, cells were fixed in 4% paraformaldehyde, blocking with Seablock (Thermo Fisher Scientific 37527) and permeabilization with 0.05% Tween-20 in PBS. Anti-CD36 (1:100; Biorad MCA2748) and anti-PHB1 (1:100; MilliporeSigma HPA0038) were applied at 4°C for 12 h and followed with secondary anti-rat Cy3 (1:200; Jackson Labs 712-166-150) and donkey anti-rabbit Alexa 488 (1:200; Life Technologies A121206). Nuclei were counterstained with DRAQ5 (1:1000; eBioscience 65088092). Images were acquired at 63X with confocal z-stack projections using Leica TCS SP5 microscope with LAS AF.

### Subcellular fractionation

Mitochondrial and cytosolic fractions were collected from tissue using the Abcam Mitochondrial/Cytosolic Protein Extraction Kit following the manufacturer’s instructions (36). Lipid raft fractions were isolated from tissue using the Bio-Rad Protein Extraction Kit (Signal). Cellular fractions were run on 10% SDS-PAGE gels. Fraction integrity was confirmed by using marker proteins for the relevant compartment (COX-IV and Mitofusin-2 for mitochondria, caveolin 1 for lipid rafts, and β-actin for cytosol) (36).

### Statistical analysis

Quantification of images was performed by using the ImageJ software (https://imagej.nih.gov/ij). Two-tailed Student’s unpaired t-tests with the Holm-Šidàk procedure (α = 0.05) were used when comparisons were only between genotypes. Two-way ANOVA followed by either Fisher’s uncorrected least significant difference tests or Tukey’s correction for multiple comparisons was used as indicated, to determine the significance of differences between sexes and between genotypes. Fisher’s least significant difference was used for analyses with only two variables (i.e., sex and genotype), while Tukey’s correction was used for analyses with three (i.e., sex, genotype, and depot). All statistical analysis and data visualization were performed using GraphPad Prism 10.

## Results

### Sex- and depot-specific effects of *Phb1* KI on white AT

To investigate the importance of Cys^69^-dependent function of PHB1, we compared body and AT mass in *Phb1*-KI mice and age/sex-matched WT mice. PHB1 protein levels were not substantially different between WT and knock-in groups (supplemental Fig. S1C, D). Body weights were comparable in *Phb1*-KI and WT females, whereas male *Phb1*-KI mice were slightly overweight (Fig. 1A). However, the ratio of VAT to body weight was higher (by ANOVA) in both *Phb1*-KI males (*P* = 0.02) and females (*P* = 0.04) compared to WT (Fig. 1A). There was no significant difference in the ratio of VAT to body weight within the same sexes between genotypes (Fig. 1A). Conversely, the ratio of SAT to body weight was lower in *Phb1*-KI male (*P* = 0.02) and female (*P* = 0.01) mice compared to respective WT mice, and there was no difference within the same sexes between genotypes (Fig. 1A).

**Fig. 1:**
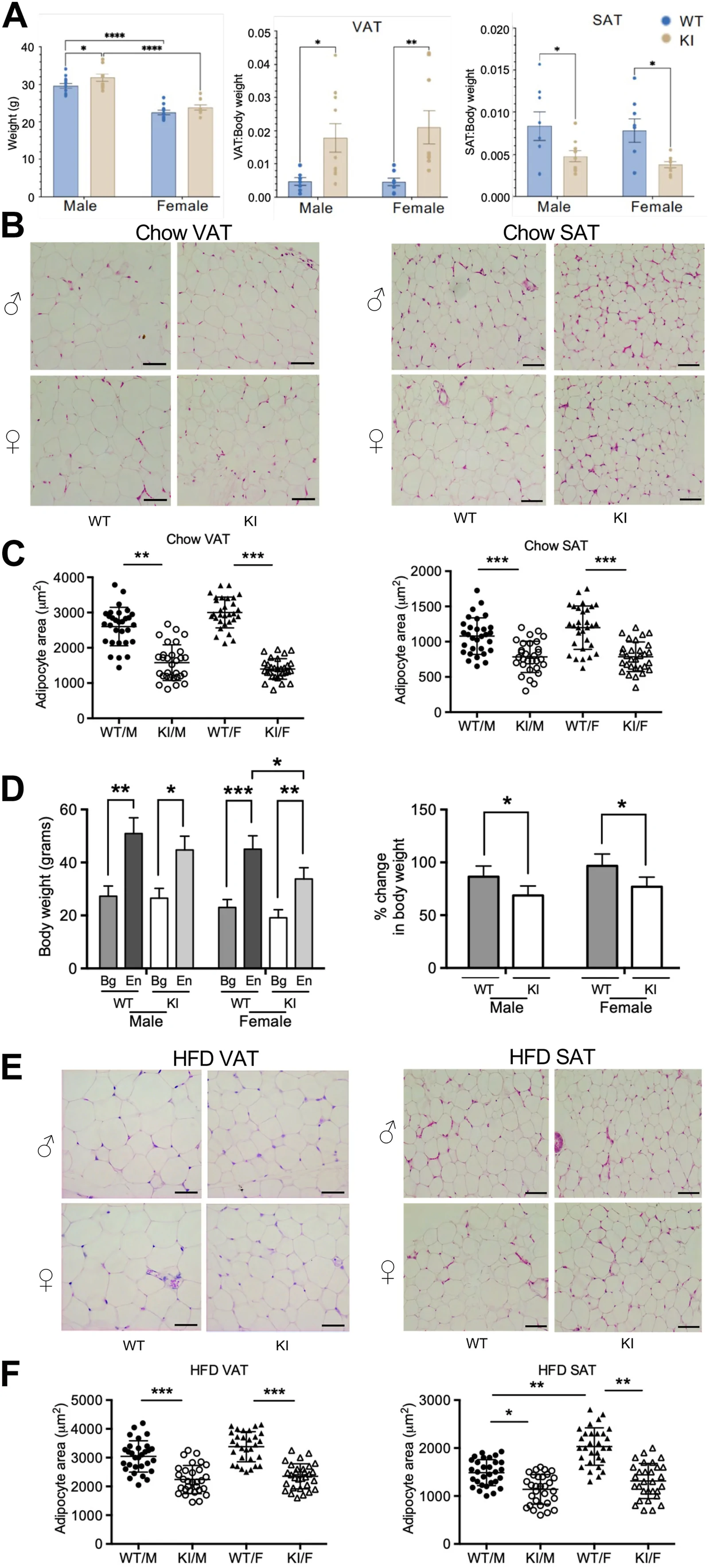
Sex- and depot-specific effects of Phb1-KI on white AT. A: Sex- and depot-specific changes in WAT mass of adult KI compared to WT mice fed chow. Plotted are whole-body weights and ratios of VAT and SAT to body. B: Representative photomicrographs showing H&E-stained sections of VAT and SAT from adult WT and KI mice fed chow. C: Quantification of SAT and VAT data from (B) showing differences in adipocyte size between WT and KI mice. n = 5–6. D: Sex-specific changes in body weight of KI compared to WT mice fed HFD for 16 weeks. Bg: body weight at the beginning of an HFD; En: body weight at the end point. E: Representative photomicrographs showing H&E-stained sections of VAT and SAT from adult WT and KI mice fed an HFD. F: Quantification of SAT and VAT data from (E) showing differences in adipocyte size between WT and KI mice. n = 5–6. Data are presented as mean ± SEM (n = 9–10, as indicated), and *P* < 0.05 by two-way ANOVA with Fisher’s uncorrected LSD test was considered significant. ∗*P* < 0.05; ∗∗*P* < 0.01; ∗∗∗*P* < 0.001; ∗∗∗∗*P* < 0.0001. The scale bar represents 100 μm. HFD, high-fat diet; LSD, least significant difference; Phb1-KI, Phb1C69A knock-in; SAT, subcutaneous adipose tissue; VAT, visceral adipose tissue.

We also performed histological analysis of WAT sections stained with H&E (Fig. 1B). It revealed a decrease in *Phb1*-KI VAT and SAT adipocyte sizes compared to WT controls (Fig. 1C). We also analyzed mice raised on a HFD, which induces obesity in WT mice. Both *Phb1*-KI males and females were found to be resistant to body weight increase compared to WT controls (Fig. 1D). The difference between females was wider than between the male mice (Fig. 1D), suggesting a sex-biased effect of the PHB1 Cys^69^ mutation. Histological analysis of WAT sections demonstrated that adipocyte size remained smaller in both VAT and SAT of *Phb1*-KI mice fed an HFD compared to WT controls (Fig. 1E, F).

### Hormone and adipokine levels in *Phb1*-KI mice

We performed analysis of serum upon chow or HFD feeding (supplemental Table S1). Ghrelin levels significantly increased (*P* < 0.01) in female *Phb1*-KI mice, whereas the opposite effect was found in the female WT mice. HFD induced a significant increase (*P* < 0.01) in insulin levels in female *Phb1*-KI mice compared to male mice, whereas in the *Phb1*-KI mice, HFD induced a similar increase in insulin levels in both male and female mice compared with respective WT mice. In the female WT mice, adiponectin levels were higher (*P* < 0.01) and leptin and resistin levels were lower (*P* < 0.05) in comparison to the male WT mice. In *Phb1*-KI mice, sex-related differences in adiponectin, leptin, and resistin levels were opposite to WT mice. The levels of resistin increased in response to an HFD in WT mice (*P* < 0.05); however, sex-related differences remain similar to WT mice but reversed in *Phb1*-Ki mice. Leptin levels were higher in female *Phb1*-KI mice, however, response to HFD for both leptin and resistin was higher in WT than in *Phb1*-KI mice. In response to HFD, GLP-1 levels significantly increased in WT males and more so in females (*P* < 0.05), whereas in the *Phb1*-KI mice, the increase and sex-related differences were blunted.

### Sex- and depot-specific effects of *Phb1* KI on adipocyte lipid homeostasis

To gain insight into the effect of *Phb1*-KI on adipocytes, we analyzed markers of lipid transport, adipogenesis, and lipolysis. CD36 is a key regulator of lipid uptake and metabolism. VAT from *Phb1*-KI mice showed a trend for higher CD36 levels in male but not in female *Phb1*-KI mice when compared with respective WT mice (supplemental Fig. S2A, B). In SAT, no significant differences in CD36 levels were observed between genotypes and sexes (supplemental Fig. S2A, B). Similarly, no significant differences were observed for LPL levels in VAT or SAT among the experimental groups (supplemental Fig. S2B). Lipin-1 is a key lipogenic effector, and differential phosphorylation of lipin-1 controls its location and function (37). Notably, lipin-1 levels showed a distinct doublet band pattern between WT and *Phb1*-KI mice, which was relatively more apparent in the female mice (supplemental Fig. S2A). Expression of adipogenic transcription factors PPARγ, C/EBPα, and C/EBPβ in SAT was not different between the genotypes (supplemental Fig. S2C). However, in VAT of male *Phb1*-KI VAT, expression of all three transcription factors was lower than for WT controls (supplemental Fig. S2C). For the females, only C/EBPβ VAT expression was significantly (*P* < 0.05) decreased in the *Phb1*-KI mice compared to WT mice (supplemental Fig. S2C). Combined, these sex- and depot-specific effects of *Phb1*-KI provide the explanation for the observed adipocyte phenotype.

### PHB1 Cys^69^ is required for its plasma membrane localization

To investigate whether the subcellular localization of PHB1 is altered in *Phb1*-KI mice, we analyzed mitochondrial, cytosolic, and lipid raft fractions of WAT by Western blotting (Fig. 2A). In both VAT and SAT, PHB1 protein levels in the mitochondrial fraction were comparable between genotypes and sexes, suggesting that the substitution of C69A does not affect mitochondrial localization of PHB1 (Fig. 2B). Similarly, no difference in PHB2 mitochondrial localization was observed (Fig. 2A, B). Through immunoblotting analysis of lipid raft fractions (Fig. 2C), a decrease in PHB1 levels was detected in *Phb1*-KI mice compared with WT mice (Fig. 2D), consistent with the established role of Cys^69^ in lipid raft localization (24).

**Fig. 2:**
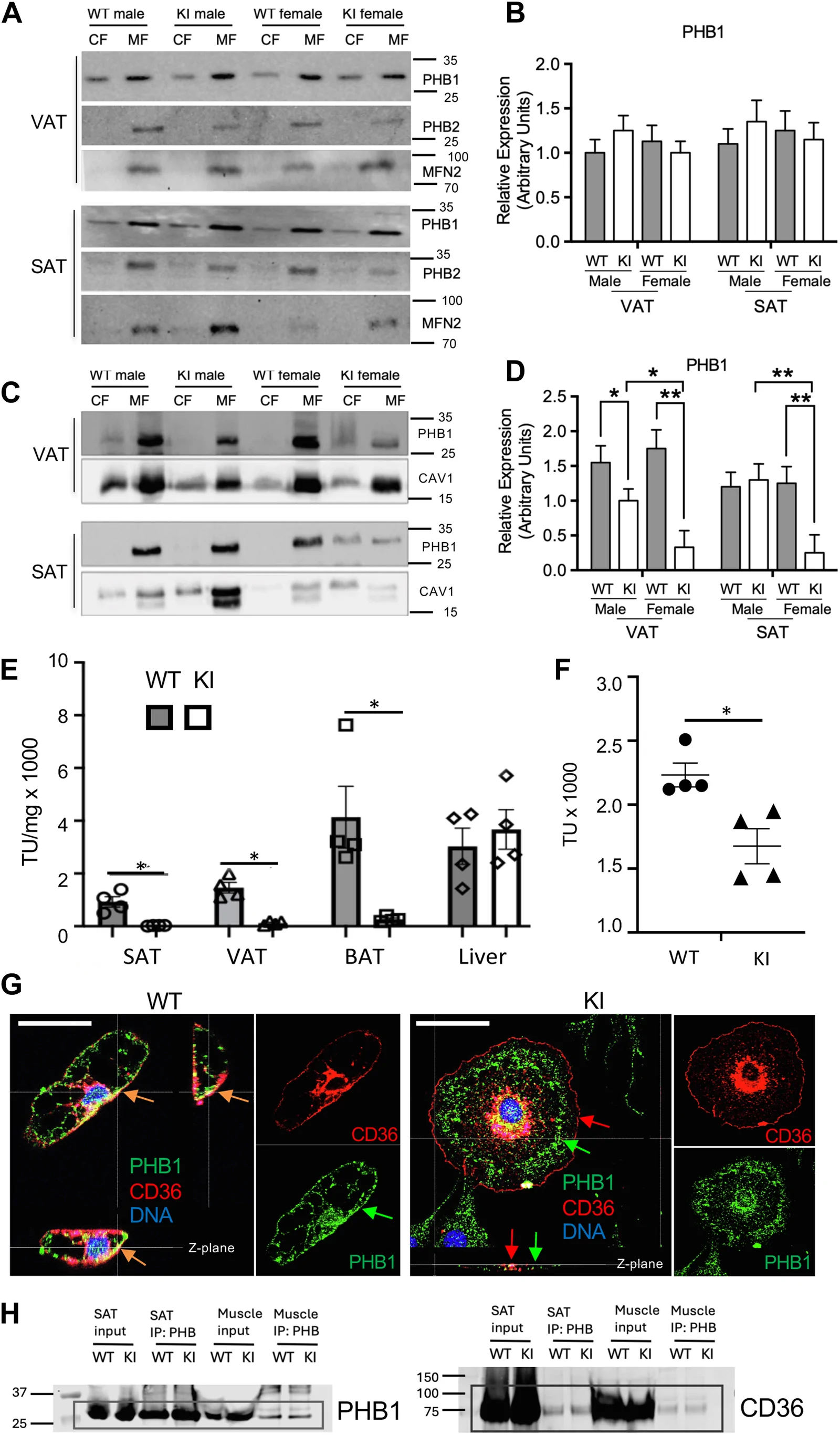
PHB1 levels in the mitochondria and plasma membranes of PHB-1 KI mice. A: Representative immunoblots showing cytosolic (CF) and mitochondrial (MF) fractions from whole VAT and SAT. Mitofusin-2 (Mfn-2) is used as a mitochondrial marker. B: Quantification of PHB1 in the mitochondrial fractions between genotypes, normalized to Mfn-2 expression. C: Immunoblots showing PHB1 expression in plasma membrane or lipid raft fractions (LRF) and nonmembrane supernatants (S) from whole VAT and SAT. Caveolin-1 (Cav-1) is used as a plasma membrane marker. D: Quantification of PHB1 expression in plasma membrane fractions between genotypes, normalized to Cav-1 expression. Data are presented as mean ± SEM (n = 3), and *P* < 0.05 by two-tailed Student’s unpaired *t* test between genotypes with the Holm-Šidàk correction was considered significant. E: CKGGRAKDC-phage was iv-injected into WT or KI male mice. After circulation for 2 h, tissues were collected, and phage transduction units (TU) were quantified (mean ± SEM). Liver shows non-specific phage accumulation serving as a positive control for equal phage particle administration. ∗*P* < 0.001 (Student’s *t* test). F: WT or KI adipocytes (7 days postadipogenesis induction in fibroblasts) were incubated with CKGGRAKDC-phage for 1 h. After cell washing, phage TU were quantified as mean ± SEM; ∗*P* < 0.001 (Student’s *t* test). G: Coimmunolocalization of PHB1 with CD36 at the plasma membrane (yellow arrows) in WT adipocytes. In Phb1-KI adipocytes, PHB1 is intracellular (green arrows), whereas CD36 is still plasmalemmal (red arrows). Analysis was performed upon 0.3 mM palmitic acid treatment for 10 min. Shown are confocal z-stack projections. Nuclei are blue. The scale bar represents 50 μm. *H*, cell lysates from WT and Phb1-KI SAT and quadriceps muscle were immunoprecipitated with PHB1 antibody and immunoblotted with anti-PHB1 and anti-CD36 antibodies. Input extract (pre-IP) and anti-PHB1 immunoblot demonstrate equal protein loading. PHB1, prohibitin-1; Phb1-KI, Phb1C69A knock-in; VAT, visceral adipose tissue.

To test the role of Cys^69^ in cell surface exposure of PHB1, we used the cyclic peptide CKGGRAKDC previously shown to home to PHB1 on adipose cells (31, 38). After iv injection of *Phb1*-KI and control mice with phage displaying CKGGRAKDC, we quantified phage accumulation in various organs. The accumulation of KGGRAKD-phage in SAT, VAT, and BAT in WT mice was reduced to a background level in *Phb1*-KI mice (Fig. 2E). We also confirmed that KGGRAKD-phage binding to and uptake by adipocytes cultured ex vivo was significantly (*P* < 0.05) reduced by *Phb1*-KI (Fig. 2F). Finally, colocalization of PHB1 with CD36 at the plasma membrane was observed for WT adipocytes, whereas their colocalization was predominantly intracellular in *Phb1*-KI adipocytes (Fig. 2G). However, the extent of PHB1-CD36 binding, measured by coimmunoprecipitation, was found to be comparable in WT and *Phb1*-KI SAT and skeletal muscle (Fig. 2H). These data indicate that Cys^69^ is not required for CD36 binding but is required for PHB1 localization to the plasma membrane and exposure on the surface of adipocytes.

### Fatty acid uptake and mobilization in WAT are defective in *Phb1*-KI mice

To determine whether the AT phenotype of *Phb1*-KI mice is due to reduced LCFA uptake, we i.v.-injected mice with a fluorophore-labeled palmitic acid, BODIPY-FL-C16, and analyzed adipocytes recovered from AT after 3 h of circulation. The deposition of C16-BODIPY was reduced in adipocytes from all three adipose depots (namely, VAT, SAT, and BAT) in *Phb1*-KI mice when compared with WT mice (Fig. 3A). The difference in BODIPY-FL-C16 accumulation was not observed in skeletal muscles between the two genotypes (supplemental Fig. S3A). Notably, the signal was higher in the livers and kidneys of *Phb1*-KI mice (supplemental Fig. S3A), which can be explained by excess circulating BODIPY-FL-C16 due to reduced WAT uptake. Confirming the role of PHB1 Cys^69^ in LCFA uptake by adipocytes, we observed that cultured *Phb1*-KI adipocytes were inefficient in C16-BODIPY deposition into lipid droplets (Fig. 3B).

**Fig. 3:**
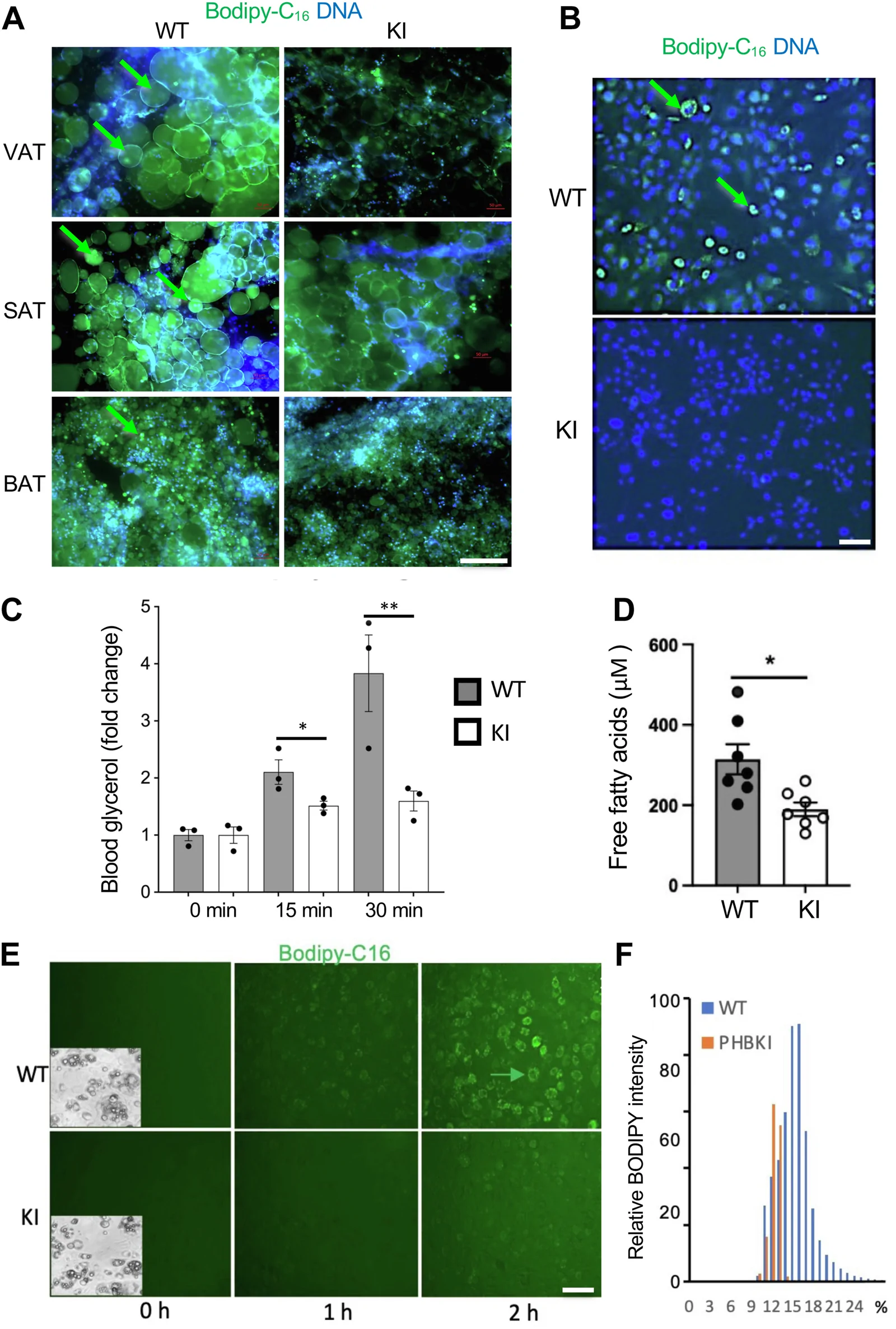
Fatty acid WAT uptake and mobilization defect in Phb1-KI mice. A: Whole mounts of VAT, SAT, and BAT 3 h after i.v. injection of BODIPY-C16 (0.1 mg/mouse) showing higher uptake (arrows) by adipocytes in WT mice compared to KI. B: Ex-vivo differentiated adipocytes were directly incubated with BODIPY-C16 (2 μM) for 15 min. Arrows: higher uptake by WT adipocytes compared to KI. C: Relative plasma glycerol concentration at indicated time points after isoproterenol injection into WT or KI females, n = 3. D: FFA in the media after isoproterenol addition to the medium of cultured WT or KI adipocytes (equal lipid droplet formation shown below) for 4 h ∗*P* < 0.05; ∗∗*P* < 0.01 (Student’s *t* test). E: BODIPY-C16 (green, arrow) transport into 3T3-L1 adipocytes (insets: brightfield differentiation confirmation) across a monolayer of WT or KI SVF cells in a transwell assay. F: Fluorescence quantification among adipocytes from (E) at 2 h. Nuclear staining: DAPI. The scale bar represents 100 μm. BAT, brown adipose tissue; Phb1-KI, Phb1C69A knock-in; SAT, subcutaneous adipose tissue; VAT, visceral adipose tissue; WAT, white adipose tissue.

To better understand the AT phenotype in *Phb1*-Ki mice, we examined lipid mobilization from AT. Glycerol mobilization upon lipolysis induction was significantly decreased in *Phb1*-KI mice in comparison with WT mice (Fig. 3C). Consistent with this observation, fatty acid release from differentiated adipocytes upon lipolysis induction was also reduced in cell culture (Fig. 3D). We also performed a transwell assay (Fig. 3E) to measure C16-BODIPY transport into cultured 3T3-L1 adipocytes across a monolayer of stromal/vascular cells (19). Unlike WT cells, *Phb1*-KI cells did not enable LCFA transcytosis into adipocytes (Fig. 3F). These data indicate that PHB1 Cys^69^, which promotes PHB1 plasma membrane exposure, is required for uptake and intercellular trafficking of lipids in AT, as well as for their systemic mobilization.

### *Phb1* KI mice display BAT abnormalities

To determine the importance of Cys^69^-linked function of PHB1 in sex-related differences in BAT, we performed histological analysis of tissues from mice fed chow. In the *Phb1*-KI mice, BAT lipid droplets were notably larger than in WT mice (Fig. 4A). BAT weight was also higher in the *Phb1*-KI mice for both males and females (Fig. 4B). In mice fed an HFD, increased accumulation of larger lipid droplets was observed in BAT (Fig. 4C). Again, lipid droplets were larger in BAT of *Phb1*-KI mice compared to WT mice (Fig. 4C). Notably, an increase in BAT weight in response to an HFD was relatively higher in female *Phb1*-Ki mice than male *Phb1*-Ki mice when compared to respective WT mice (Fig. 4D). Histological analysis revealed hepatic steatosis in *Phb1*-KI mice, which was more pronounced in females (supplemental Fig. S3B). These data suggest that reduced retention of triglycerides in WAT might have contributed to their increased deposition into BAT and the liver.

**Fig. 4:**
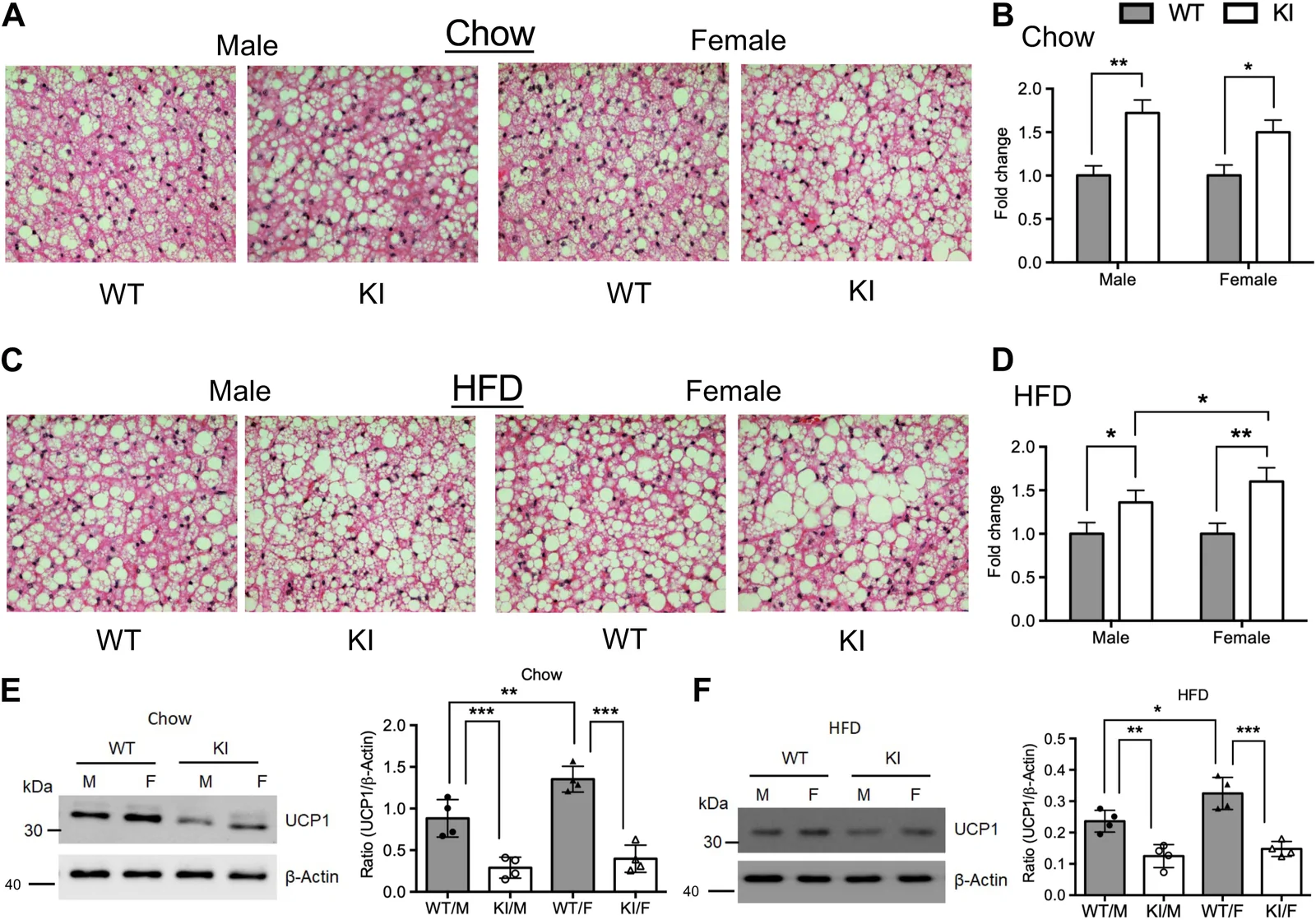
Phb1-KI effect on BAT. A: Representative H&E-stained sections of interscapular BAT from WT and KI mice fed chow. B: Weights of interscapular BAT from KI and WT male (M) and female (F) mice fed chow. The scale bar represents 50 μm. C: Representative H&E-stained sections of interscapular BAT from WT and KI mice fed HFD. D: Weights of interscapular BAT from KI and WT mice fed HFD. E: Immunoblot showing UCP1 expression in BAT of WT and KI male (M) and female (F) mice, and histogram showing quantification of protein band densities normalized to beta-actin. ∗*P* < 0.05; ∗∗*P* < 0.01; ∗∗∗*P* < 0.001 (two-way ANOVA). n = 10. The scale bar represents 50 μm. BAT, brown adipose tissue; HFD, high-fat diet; Phb1-KI, Phb1C69A knock-in.

Increased lipid accumulation in BAT of *Phb1*-KI mice prompted us to analyze the expression of the key thermogenic factor, UCP1. Immunoblot analysis of BAT lysates showed a significant (*P* < 0.01) decrease in UCP1 levels in male and female *Phb1*-Ki mice fed chow compared to respective WT mice (Fig. 4E). Moreover, a significant decrease in UCP1 level was found in BAT from male and female *Phb1*-Ki mice in response to an HFD by immunoblotting compared with respective WT mice (Fig. 4F).

### Brown adipocytes of *Phb1*-KI mice have defective mitochondria

PHB1 is required for normal mitochondrial activity (39), and the observed reduction in UCP1 expression in *Phb1*-KI mice suggested a role of Cys^69^-linked function in BAT mitochondria. To investigate if the PHB1 Cys^69^ mutation affects mitochondrial function in a sex-specific manner, we analyzed their structure in BAT by TEM. BAT from the female WT mice displayed larger mitochondria and better cristae packaging than their male counterpart (Fig. 5A). Notably, an opposite trend was observed for BAT of female and male *Phb1*-KI mice (Fig. 5A), which was reflected in cellular mitochondrion numbers (Fig. 5B). Although lipid droplet size increased in BAT from both male and female *Phb1*-KI mice (Fig. 5A, B), the female *Phb1*-KI mice showed smaller mitochondria and relatively poor cristae organization compared to the male *Phb1*-KI mice (Fig. 5A). On an HFD, lipid droplet size substantially increased and mitochondrial cristae organization further deteriorated in the *Phb1*-KI mice, with reduced cristae number and swollen inter-cristae space observed (Fig. 5C). Moreover, on an HFD, the male *Phb1*-KI mice displayed better BAT mitochondrial organization than the female *Phb1*-KI mice; however, this was opposite between male and female WT mice (Fig. 5C, D). These data suggested the importance of a PHB1 Cys^69^-linked function in maintaining a sex-related differences in mitochondrial function. We also analyzed liver, adrenocortical, and testicular Leydig cells from *Phb1-KI* mice by TEM. We did not detect mitochondrial abnormalities in these tissues (supplemental Fig. S4A) despite increased accumulation of lipid droplets in these cell types. This indicates that the mitochondrial cristae abnormality in *Phb1-KI* mice is tissue-selective.

**Fig. 5:**
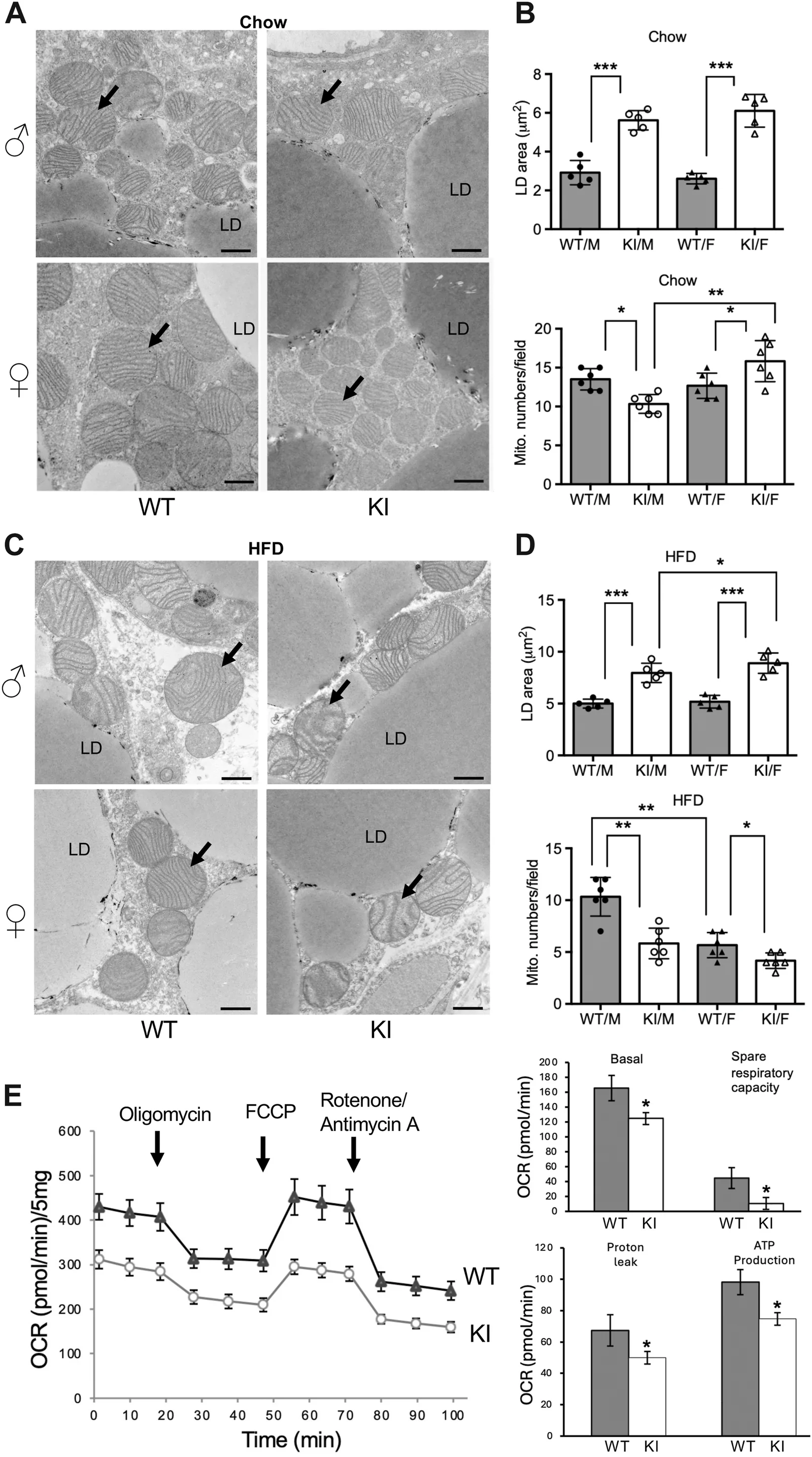
Phb1-KI BAT adipocyte mitochondrial defect. A: Representative TEM micrographs of BAT from KI and WT mice fed chow with mitochondria (arrow) differences indicated. B: Quantification of TEM analysis of BAT showing changes in lipid droplet (LD) area and mitochondrion number per field in KI mice versus WT male (M) and female (F) mice fed chow. C: Representative TEM micrographs of BAT from KI and WT mice fed HFD. D: Quantification of TEM analysis of BAT showing changes in lipid droplet area and mitochondrion number per field in KI mice versus WT mice fed HFD. n = 5. ∗*P* < 0.05; ∗∗*P* < 0.01; ∗∗∗*P* < 0.001 (two-way ANOVA). TEM magnification: 25,000X. E: XF Cell Mito Stress assay on BAT from 2-month-old chow-fed males (n = 12). Upon successive treatment with oligomycin, FCCP, and rotenone/antimycin A, OCR (mean ± SD) reveals lower mitochondrial respiration in KI BAT. ∗*P* < 0.05 (Student’s *t* test). Basal and spare respiratory capacity, as well as ATP production and proton leak, are plotted on the right. BAT, brown adipose tissue; OCR, oxygen consumption rate; Phb1-KI, Phb1C69A knock-in; TEM, transmission electron microscope.

We directly measured mitochondrial function by Seahorse XF Cell Mito Stress Assay. Analysis of BAT from 2-month-old males raised on chow revealed a significant decrease in basal and induced OCR of KI mice (Fig. 5E). Spare respiratory capacity and ATP production was also reduced compared to WT mice (Fig. 5E). Consistent with reduced UCP1 expression, BAT from *Phb1*-KI mice displayed lower proton leak indicating a defect in uncoupling (Fig. 5E). To determine if the mitochondrial abnormalities are specific to brown adipocytes, we analyzed primary tissue-cultured ear-derived fibroblasts. Immunofluorescence revealed that the density of mitochondria expressing COXIV and PHB1 expression and colocalization with mitochondria was comparable for WT and KI cells (supplemental Fig. S4B). In fibroblasts, Seahorse XF Cell Mito Stress Assay measurement of lower basal and induced OCR revealed a significant (*P* < 0.01) decrease in mitochondrial respiration of *Phb1*-KI cells (supplemental Fig. S4C). Notably, extracellular acidification rate was significantly higher for *Phb1*-KI cells (supplemental Fig. S4C). This indicated that the cells with PHB1 Cys^69^ mutation are inefficient in oxidative phosphorylation and compensate by becoming more reliant on glycolysis. We also performed a Palmitate Oxidation Stress Test on BAT and skeletal muscle. Maximal respiration was found to be lower in both BAT and quadriceps from *Phb1*-Ki compared with (supplemental Fig. S4D). This indicated that a defect in burning extracellular fatty acids is not adipocyte specific. However, a difference in magnitude was observed between *Phb1*-KI mice and in BAT and muscle samples, which was relatively larger in BAT. These data suggest that the importance of Cys^69.^ is tied to fatty acid metabolism and is not uniform across tissues.

### Glucose metabolism upregulation in *Phb1*-KI mice

Next, we performed GTT and ITT to determine glucose homeostasis in *Phb1-KI* mice. In the GTT, an improvement in glucose clearance was observed in the *Phb1*-KI mice compared to WT mice (Fig. 6A). In the ITT, a significant difference in insulin sensitivity (*P* < 0.05) was found between WT male and female mice (Fig. 6B). However, this sex-specific difference was negated in *Phb1*-KI mice, where higher insulin sensitivity was observed for both males and females (Fig. 6B). A similar GTT trend was observed between male and female WT and *Phb1*-KI mice raised on HFD (Fig. 6C). On an HFD, both male and female *Phb1*-KI mice displayed increased insulin sensitivity when compared with their respective male and female counterparts (Fig. 6D). Moreover, male and female WT mice showed a significant difference in their insulin sensitivity, which was not observed between male and female *Phb1*-KI mice (Fig. 6D). While fasting glucose was not affected (Fig. 5E), fasted *Phb1*-KI mice had lower circulating insulin levels (Fig. 6F). They also had lower homeostatic model assessment of insulin resistance, an approximator of insulin resistance (Fig. 6G). Importantly, *Phb1*-KI mice had a significantly higher lactate concentration in plasma (Fig. 6H). These data, consistent with the Seahorse results (supplemental Fig. S4C, indicate that the absence of Cys^69^ on PHB1 enables increased glucose utilization at least in part through glycolysis activation.

**Fig. 6:**
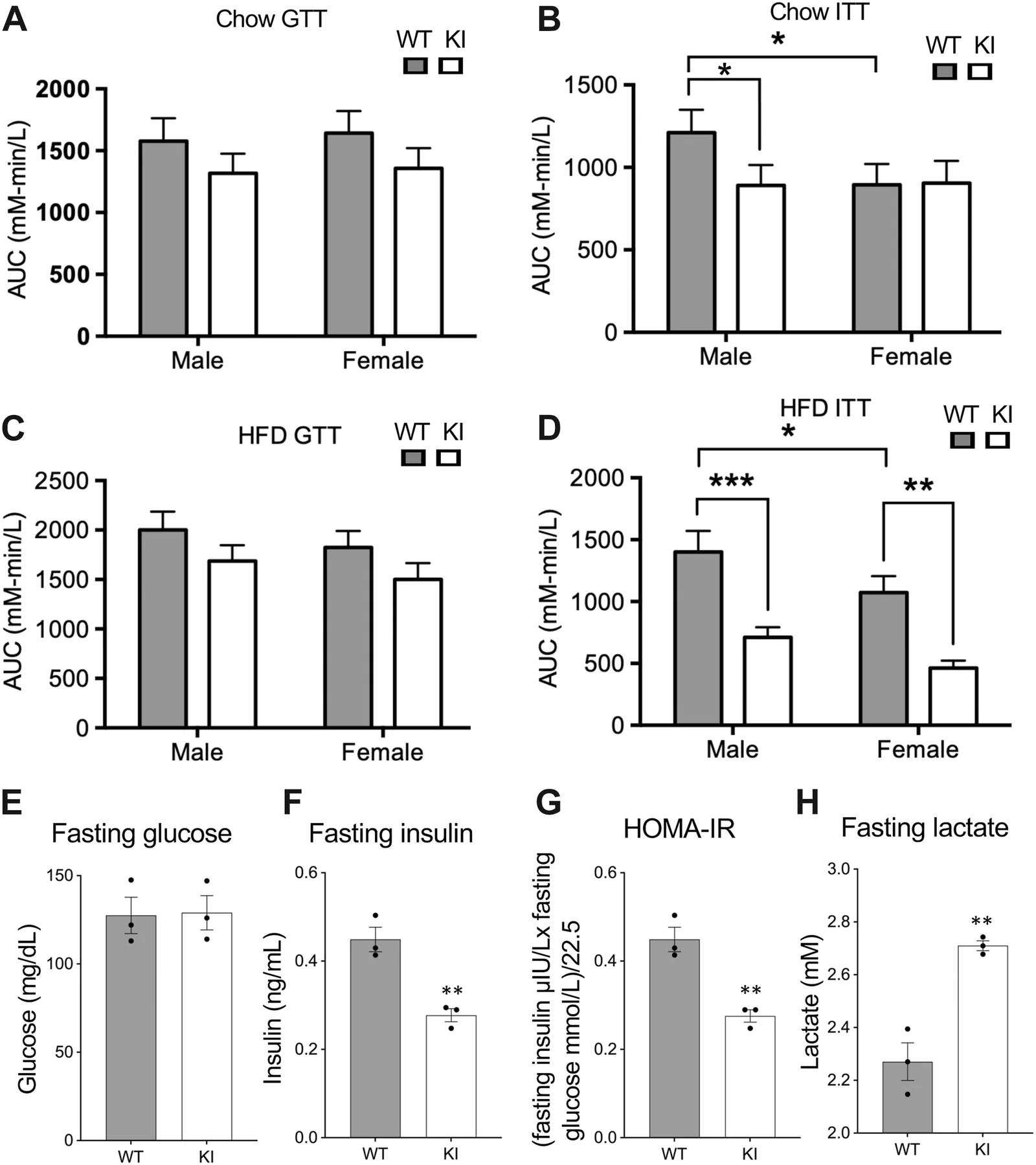
Phb1-KI mice display improved glucose metabolism. A: GTT area under the curve (AUC) in adult male and female WT and KI mice fed chow. B: ITT AUC in adult male and female WT and KI mice fed chow. C: GTT AUC in adult male and female WT and KI mice fed HFD. D: ITT AUC in adult male and female WT and KI mice fed HFD. In (A–D), n = 5–6, ∗*P* < 0.05, ∗∗*P* < 0.01, ∗∗∗*P* < 0.001 (two-way ANOVA). E: Fasting plasma glucose in adult WT and KI males fed chow. F: Fasting plasma insulin in adult WT and KI males fed chow. G: HOMA-IR, calculated as (fasting insulin μIU/L x fasting glucose mmol/L)/22.5, in adult WT and KI males fed chow. H: Fasting plasma lactate in adult WT and KI males fed chow. In (E–H), ∗*P* < 0.05, ∗∗*P* < 0.01 (Student’s *t* test). Phb1-KI, Phb1C69A knock-in; GTT, glucose tolerance test; HFD, high-fat diet; HOMA-IR, homeostatic model assessment of insulin resistance; ITT, insulin tolerance test.

### AT responds differently to gonadectomy in male and female *Phb1*-KI mice

Our previous studies using AT-specific transgenic and KO mouse models of PHB1 have provided evidence suggesting a relationship between PHB1 and sex steroids in sex-related differences in AT (13, 20, 21). To investigate a potential role of PHB1 Cys^69^, we examined the effect of surgical gonadectomy on AT phenotype in *Phb1*-KI mice. Measurement of adipokines in their serum samples showed that gonadectomy resulted in significant decrease in leptin and resistin levels in the female *Phb1*-KI mice compared with male mice, whereas an opposite effect was observed on adiponectin levels in *Phb1*-KI mice (Supplemental Table S1). In addition, gonadectomy affected mitochondrial size and cristae organization, as well as lipid droplet size in BAT from *Phb1*-KI mice (Fig. 7A). The difference in mitochondrial phenotype, such as smaller mitochondria and poor cristae organization, was more apparent between females than males when compared with their respective mice (Fig. 7B). Taken together, these data indicate the importance of Cys^69^ in sex-selective metabolic functions of PHB1.

**Fig. 7:**
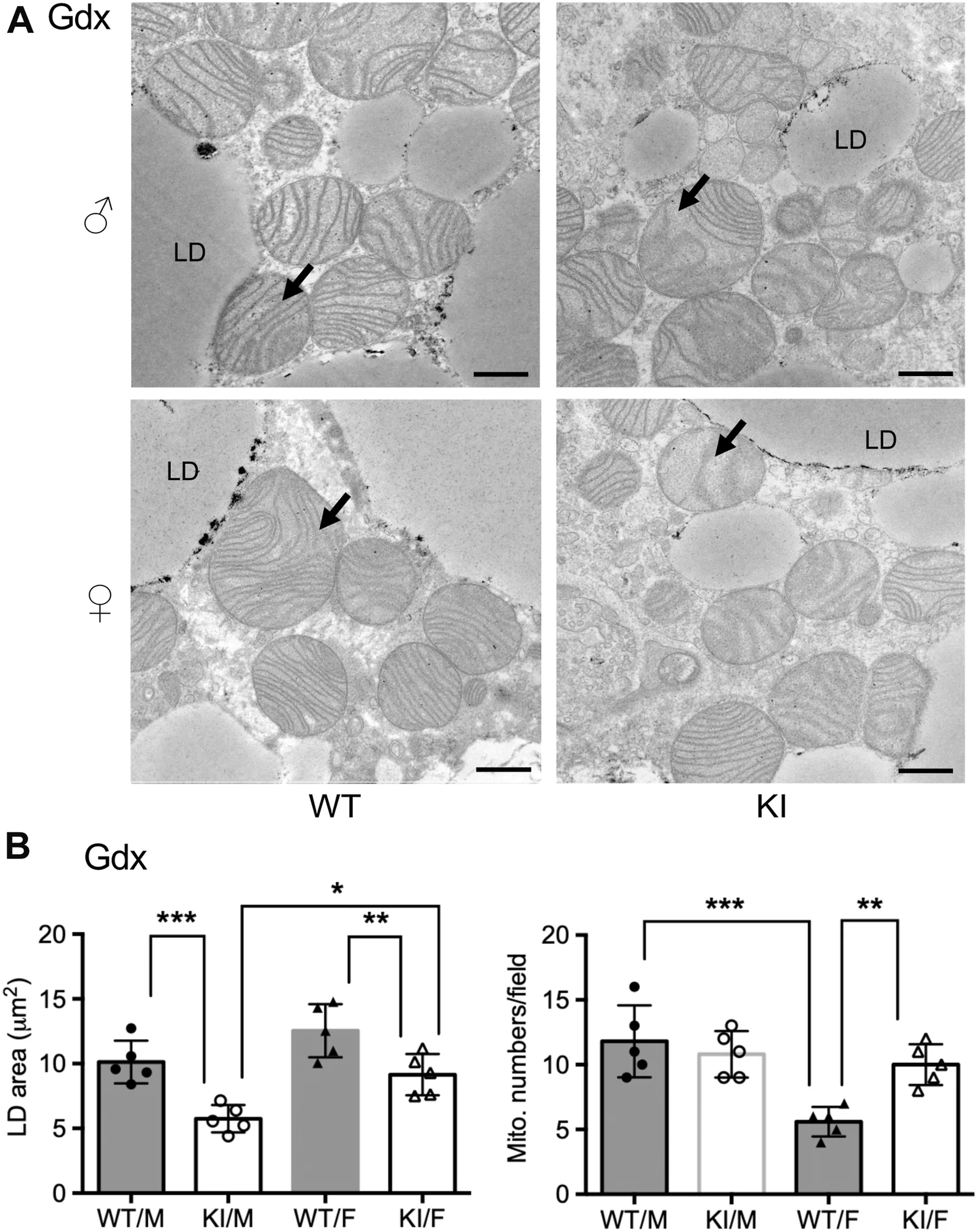
Response to gonadectomy in male and female *Phb1*-KI mice. A: Representative TEM micrographs (25,000X) of BAT from KI and WT mice fed chow after Gdx, with mitochondria (arrow) differences indicated. B: Quantification of TEM analysis of BAT showing changes in lipid droplet (LD) area and mitochondrion number per field in KI mice versus WT mice fed chow after Gdx. n = 5. ∗*P* < 0.05; ∗∗*P* < 0.01; ∗∗∗*P* < 0.001 (two-way ANOVA). BAT, brown adipose tissue; Phb1-KI, Phb1C69A knock-in; TEM, transmission electron microscope.

## Discussion

Our previous studies using AT-specific genetic gain- and loss-of-function approaches in mouse models have established a crucial role of PHB1 in AT biology and metabolism (20, 21). However, the mechanism of PHB1 function has remained unclear. In this study, using a novel *Phb1*-KI^C69A^ mouse model, we demonstrate the importance of PHB1 Cys^69^. Our findings indicate adipocyte compartment-specific functions of PHB1 Cys^69^. They also demonstrate the functional importance of Cys^69^ for PHB1 sex-biased functions in BAT and WAT. *Phb1*-KI sex-related abnormalities in AT structure and function, as well as associated glucose and lipid homeostasis, were altered in response to gonadectomy and HFD feeding. This manifestation of sex-related differences in AT and metabolic phenotype in *Phb1*-KI mice indicates that Cys^69^ has multifaceted roles in the regulation of sex-related functions of PHB1.

Overall, Cys^69^ KI causes a phenotype similar to that described for the complete PHB1 KO(17, 20), although it does not affect the level of PHB1 protein expression. This emphasizes the importance of Cys69 for the pleotropic PHB1 functions. Our findings provide strong evidence that PHB1 Cys^69^ plays both mitochondrial and extra-mitochondrial plasma membrane-associated roles in BAT and WAT biology, respectively. They substantially advance our understanding of the mechanism through which PHB1 regulates cell metabolism. The opposite effect of the *Phb1*-KI on lipid droplet size in white and brown adipocytes may be due to cell type-specific roles of PHB1. This could be related to lipid handling in white and brown adipocytes (i.e., lipid storage and mobilization versus. oxidation), as well as to a substantial difference in their mitochondrial contents. PHB1 exposure on the cell surface in AT has been uncovered through studies based on the synthetic peptide CKGGRAKDC, mimicking a segment of ANX2, that homes to WAT (26, 31, 38). Our previous studies (24) suggest that S-acylation of Cys^69^ is required for PHB1 exposure on the cell surface. This function explains the reduced lipid accumulation in white adipocytes of *Phb1*-KI mice.

PHB1 has been identified as a cysteine-oxidized mitochondrial protein in relation to oxidative stress-induced mitochondrial dysregulation in hepatocytes (36). Consistent with this notion, *Phb1*-KI brown adipocytes display mitochondrial abnormalities. The larger lipid droplets in *Phb1*-KI adipocytes are indicative of defective beta-oxidation, which is confirmed by palmitate oxidation stress test. Of note, previously we have found that PHB1^Y114F^ mutant macrophages display decreased OCR, and that Cys^69^-linked cell membrane-related function facilitates phosphorylation of PHB1 at Tyr114 residue (24). As a cysteine residue could potentially undergo different PTMs (e.g., palmitoylation and nitrosylation) in a mutually exclusive manner and PHB1-Tyr114 phosphorylation creates binding site for SH2 domain containing proteins (e.g., Shp1 phosphatase and cytoplasmic tyrosine kinase Lyn and Fyn) (40, 41), it is likely that these conserved sites in PHB1 have cell type-specific functions. Our metabolic analysis of cultured cells demonstrates that fibroblasts without PHB1 Cys^69^ also have reduced respiration, compensated for by glycolysis upregulation. It is possible that this may involve cell signaling changes (apart from direct effect on mitochondria) due to altered Tyr114-linked function as observed in our previous study in macrophages expressing mutant-PHB1Y114F (42), which work downstream Cys^69^-linked membrane signaling function (24). Therefore, it is likely that metabolism is affected differently by the Cys^69^ mutation in distinct cell types. *Phb1*-KI brown adipocytes may display the marked mitochondrial abnormalities due to their outstandingly high metabolic activity and unique thermogenic mitochondrial function. The mechanisms regulating PHB1 Cys^69^ allocation toward the cell surface LCFA transport versus mitochondrial support remain to be determined. The systemic phenotype of *Phb1*-KI mice is likely mediated by organs other than AT. Hepatic metabolism, potentially contributing to the systemic phenotype, was not explored. The mitochondrial function of PHB1 may also explain the significant decrease in fasting insulin level in *Phb1*-KI mice compared with WT mice, as mitochondria play an important role in insulin secretion (43) by pancreatic beta cells, also relying on PHB1 function (44). Moreover, an increase in insulin sensitivity as observed in *Phb1*-KI mice may relate to the membrane signaling function of PHB1.

A limitation of this study is that the knock-in model is not cell type-specific. The combined effect of tissues other than AT on the metabolic phenotype of *Phb1*-KI mice complicates data interpretation. However, our complementary in vivo and ex vivo analyses, reinforced by previous studies on adipocyte-specific manipulation of PHB1 (20, 21), indicate the importance of PHB1 Cys^69^ in adipocyte metabolism. It should be emphasized that Cys^69^ is a PTM site, which is likely dynamically regulated in a cell type- and context-specific manner like phosphorylation of PHB1 (16). Therefore, the effect of Cys^69^ mutation is likely to also be tissue- and context-specific. On the other hand, this whole-body model provides a foundational insight into the versatility of PHB1 cysteine functions at the systemic level.

In summary, this study establishes the importance of PHB1’s Cys^69^-linked functions in adipocytes, at least some of which are driven by sex hormones. We conclude that its function in white adipocytes is important for LCFA transport, whereas in brown adipocytes and myocytes it is also important for mitochondrial function in fatty acid oxidation.

## Data availability

Data generated and resources used in the study are available from the corresponding authors upon request.

## Supplemental data

This article contains supplemental data.

## Conflict of interest

R. P. and W. A. are founders and equity shareholders of PhageNova Bio. R. P. serves as the Chief Scientific Officer and paid consultant of PhageNova Bio. R. P. and W. A. are founders and equity shareholders of MBrace Therapeutics and serve as paid consultants. R. P. and W. A. have sponsored Research Agreements (SRAs) with PhageNova Bio and MBrace Therapeutics, which are managed in accordance with the established institutional conflict-of-interest policies of Rutgers, The State University of New Jersey.
