# Supplementary material for: Prohibitin-1 cysteine-69 plays a sex-dependent role in fatty acid metabolism
Source: J Lipid Res. 2026 Sep 23;67(11):101158. doi: 10.1016/j.jlr.2026.101158 (PMC13692682; doi:10.1016/j.jlr.2026.101158)
Supplement: Supplementary file 1 [file mmc1.pdf]

Analyte	WT						KI					
	Normal		Gdx		HFD		Normal		Gdx		HFD	
	Male	Female	Male	Female	Male	Female	Male	Female	Male	Female	Male	Female
Ghrelin	3.00 ± 0.63	3.09 ± 0.62	3.08 ± 0.76	6.16** ± 0.89	1.98 ± 0.34	1.90 ± 0.32	3.37 ± 0.57	3.07 ± 0.78	2.96 ± 0.67	3.20† ± 0.84	3.18† ± 0.63	5.11**,†† ± 0.75
GIP	0.17 ± 0.06	0.21 ± 0.07	0.06 ± 0.007	0.02** ± 0.005	0.08 ± 0.003	0.16* ± 0.04	0.08 ± 0.003	0.22* ± 0.03	0.06 ± 0.008	0.07† ± 0.009	0.12 ± 0.06	0.04* ± 0.003
GLP-1	0.01 ± 0.001	0.03 ± 0.002	0.009 ± 0.001	0.01 ± 0.001	0.04† ± 0.003	0.11** ± 0.04	0.05 ± 0.006	0.01* ± 0.002	0.02†† ± 0.002	0.04 ± 0.003	0.09 ± 0.005	0.02** ± 0.001
Glucagon	0.06 ± 0.004	0.14 ± 0.012	0.05 ± 0.006	0.05 ± 0.005	0.10 ± 0.07	0.10 ± 0.07	0.22 ± 0.014	0.10** ± 0.011	0.16 ± 0.06	0.23* ± 0.03	0.09 ± 0.012	0.08 ± 0.013
Insulin	1.21 ± 0.09	1.98 ± 0.09	1.47 ± 0.08	1.02* ± 0.08	7.92††† ± 1.00	16.83** ± 1.86	2.44 ± 0.17	2.48 ± 0.18	1.76 ± 0.16	8.22***,††† ± 0.83	11.90† ± 1.81	10.82† ± 0.09
Adiponectin	18.94 ± 2.21	27.71** ± 2.27	12.24 ± 1.58	16.27 ± 1.59	9.28 ± 1.12	12.34 ± 1.37	21.25 ± 2.34	8.19** ± 0.99	30.96†† ± 3.42	17.29* ± 1.87	33.47††† ± 3.89	25.24† ± 3.12
Leptin	0.94 ± 0.09	0.62* ± 0.08	3.20 ± 0.04	2.17* ± 0.03	11.27 ± 1.36	14.85 ± 1.34	0.99 ± .096	1.97*, ††† ± 0.99	3.88 ± 0.40	4.57†† ± 0.42	9.54 ± 0.86	9.30† ± 0.83
Resistin	8.96 ± 0.99	4.17* ± 0.07	7.89 ± 0.98	4.84* ± 0.08	25.63 ± 2.79	15.98* ± 1.17	5.24† ± 0.07	16.33***,††† ± 1.78	8.04 ± 0.99	10.02††† ± 1.35	7.47††† ± 1.00	20.95*** ± 1.87

Supplemental Table 1 Plasma hormone and adipokine levels.
Proteins (ng/ml) were measured by using the mouse diabetes panel (Bio-Rad). Comparisons are sex-matched chow-fed (normal) vs. normal, Gdx vs. Gdx, and HFD vs. HFD between wild-type and *Phb1*-KI mice. * P < 0.05; ** P < 0.01; *** P < 0.001 indicates significant differences between sexes (within genotype, including similar experimental manipulations), † P < 0.01; †† P < 0.01; ††† P < 0.001 indicates significant differences between genotypes (within sex, including similar experimental manipulations) by Student *t* test.

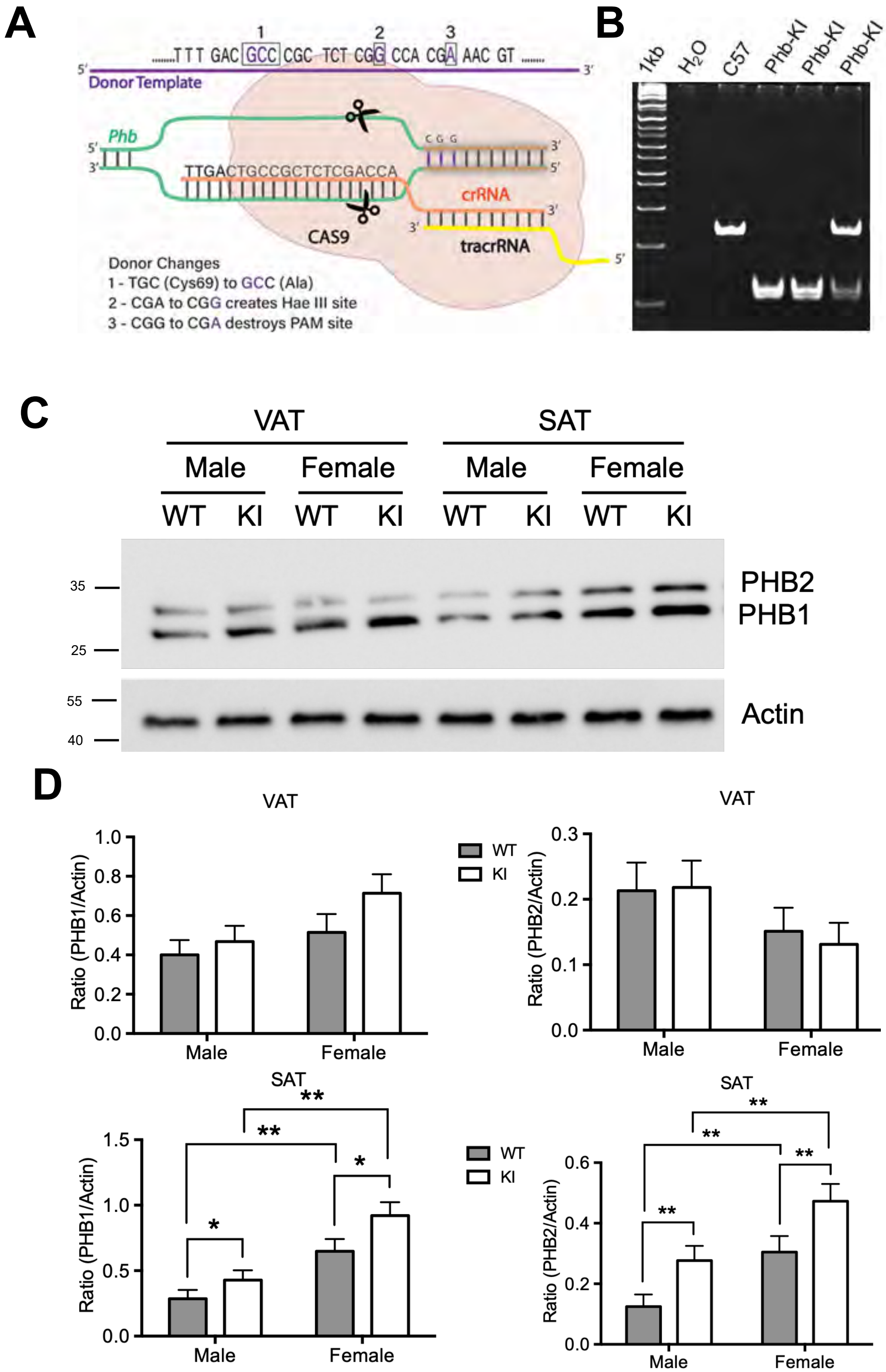

Supplemental Figure 1 Supplemental Figure 1. *Phb1* KI does not affect the cellular levels of PHB1. (**A**) The CRISPR-Cas9 strategy for PHB1 KI generation. (**B**) Examples of identification of homozygous PHB1 KI (high mobility band only) and heterozygous PHB1 KI (two bands) mice by PCR. (**C**) Immunoblots showing PHB1 and PHB2 levels in whole-tissue lysates from VAT and SAT, compared by genotype and by sex (n = 3). β-actin is used as a loading control. (**D**) Quantification of PHB1 and PHB2 protein levels in C normalized to β-actin level as a loading control. Data are presented as mean ± SEM (n=3). For protein quantification, two-way ANOVA with Tukey's multiple comparisons was used. P<0.05 **P<0.01; ***P<0.001.

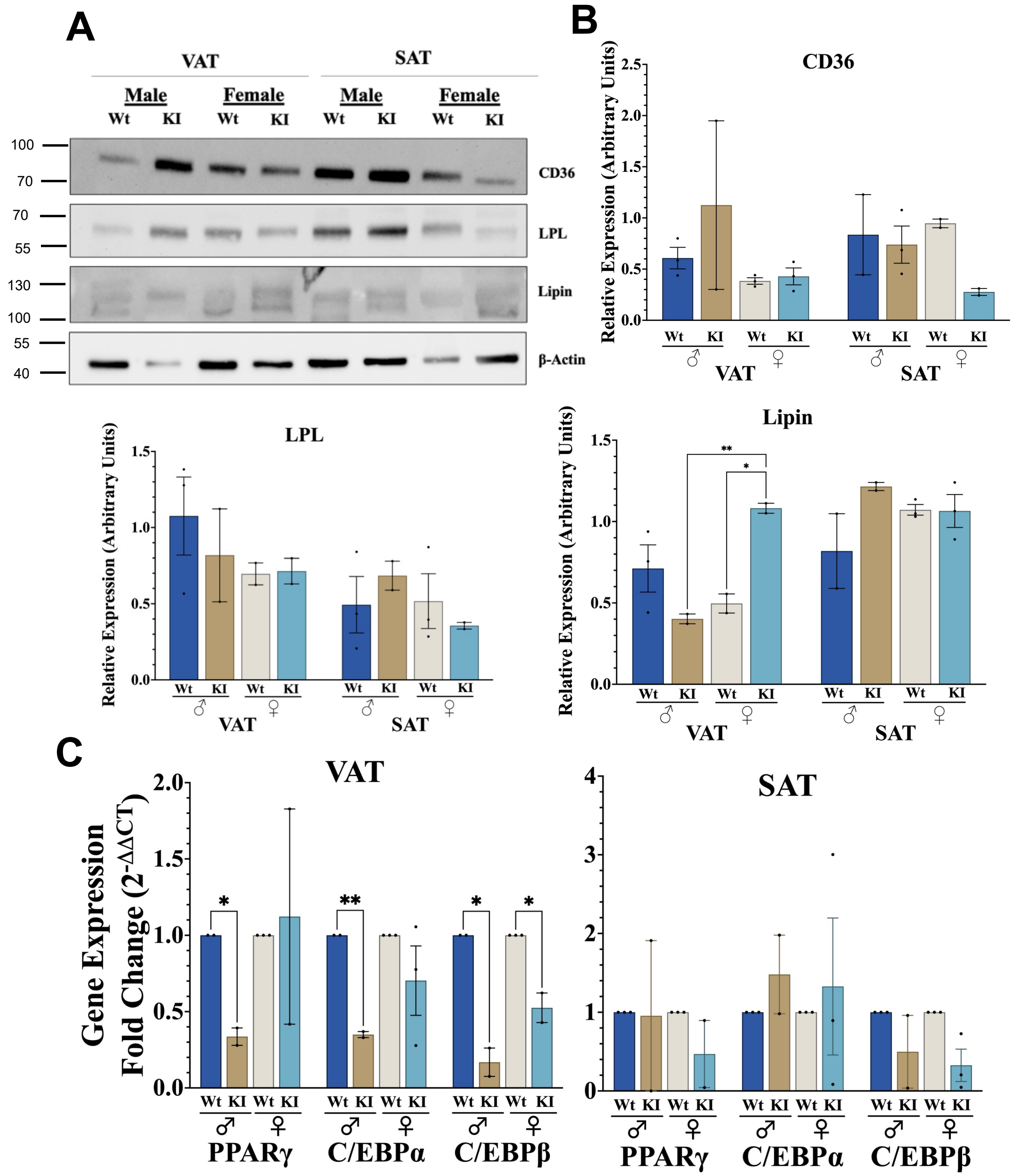

Supplemental Figure 2 *Phb1* KI does not affect key lipid metabolism effectors. (**A**) Immunoblots of VAT and SAT from WT and KI mice. (**B**) Quantification of CD36, lipid protein lipase (LPL), and lipin-1 protein expression from immunoblot (A), normalized to β-actin level as a loading control. (**C**) mRNA expression of *Pparγ*, *C/ebpα*, and *C/ebpβ*, normalized to *18s* expression. Data: mean± SEM (n = 3); *P<0.05 *P<0.01 by two-tailed Student's unpaired *t*-test with the Holm-Šidàk correction for multiple comparisons. Primers used were as follows.

18S: 5'AACTTTCGATGGTAGTCGCCGT / 5'TCCTTGGATGTGGTAGCCGTTT;
PHB1: 5'GATTCCGTGGCGTACAGGA / 5'GCGGCAGTCAAAGATAATTGGTT;
PHB2: 5'ATCCGTGTTCACCGTGGAAG / 5'CCCGAATGTCATAGATGATGGG;
PPARγ: 5'CCCACCAACTTCGGAATCAG / 5'AATGCGAGTGGTCTTCCATCA;
C/EBPα: 5'AGTCGGTGGACAAGAACAGC / 5'ACTCCAGCACCTTCTGTTGC;
C/EBPβ: 5'CAAGCTGAGCGACGAGTACA / 5'CAGCTGCTCCACCTTCTTCT.

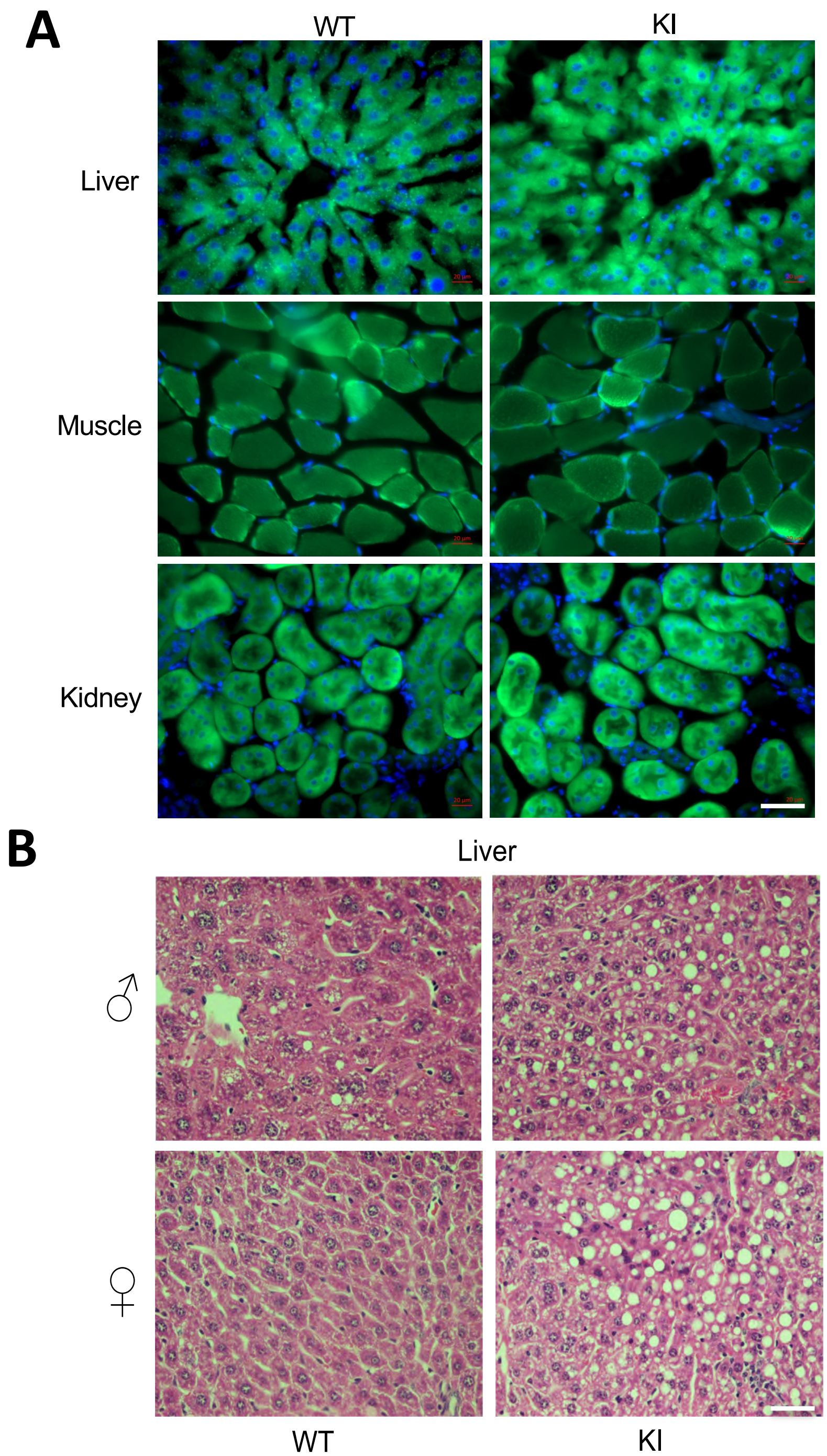

Supplemental Figure 3. The effect of *Phb1* KI on lipid accumulation. (**A**) BODIPY-C_{16} uptake in liver, skeletal muscle (quadriceps), and kidney is comparable in WT and KI mice 3 hours after i.v. injection of BODIPY-C_{16} (0.1 mg/mouse). (**B**) Representative H&E-stained liver sections reveal hepatic steatosis in KI mice. Scale bar: 50 μm.

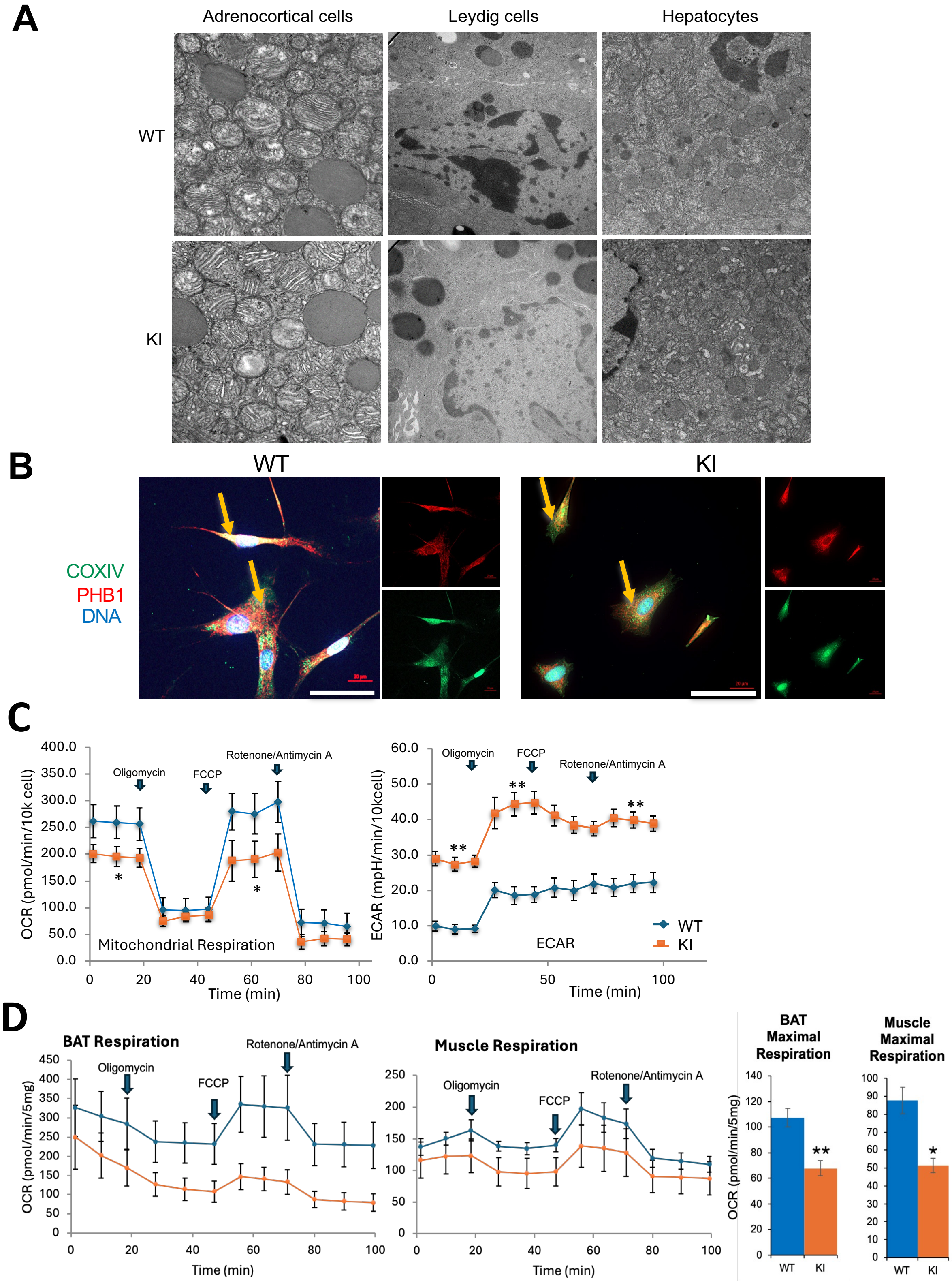

Supplemental Figure 4. *Phb1* KI effect on mitochondria in non-adipose tissues. (**A**) Representative TEM micrographs of adrenocortical cells, Leydig cells, and hepatocytes from KI and WT mice. Magnification: 25,000X. (**B**) Immunofluorescence on cultured KI and WT fibroblasts showing comparable density of mitochondria (marked by COXIV, green) and comparable PHB1 expression (red) and co-localization with mitochondria (yellow, arrows). Nuclear staining: DAPI. Scale bar, 50μm. (**C**) XF Cell Mito Stress assay on cultured KI vs WT fibroblasts, cultured in DMEM / 10% FBS, based on successive treatment with oligomycin (1μM), FCCP (1μM), and rotenone/antimycin A (0.5μM). OCR reveal lower basal and induced mitochondrial function in KI cells. ECAR reveals increased glycolysis in KI cells. Data (mean ± SD) were normalized to plated cell density, quantified based on nuclear staining with DAPI. (**D**) BAT and quadriceps muscles from male WT and PHB-KI 10-week-old mice fed chow (n=3) were subjected to Mito-stress assay with 200uM of Palmitate-BSA in the medium. *P<0.05; **P<0.001; (Student's t-test).
